# Salvaging the Last Dialysis Access: Cross-Neck Cephalic Arch-to-Contralateral Internal Jugular Vein Bypass for Chronic Total Central Venous Occlusion

**DOI:** 10.3400/avd.cr.26-00012

**Published:** 2026-05-01

**Authors:** Erwin Hadi Chandra, Dedy Pratama, Raden Suhartono, Patrianef Darwis, Alexander Jayadi Utama

**Affiliations:** Division of Vascular and Endovascular Surgery, Department of Surgery, Faculty of Medicine, University of Indonesia, Cipto Mangunkusumo Hospital, Jakarta, Indonesia

**Keywords:** central venous occlusion, hemodialysis access, venous hypertension, cross-neck venous bypass, access salvage

## Abstract

A 65-year-old hemodialysis patient with chronic total occlusion (CTO) of the right brachiocephalic vein and concurrent dialysis access–induced steal syndrome presented with venous hypertension and digital-brachial index (DBI) of 0.42. The brachiocephalic arteriovenous fistula was his sole remaining vascular access. After failed endovascular recanalization, simultaneous extra-anatomic cross-neck cephalic arch-to-contralateral internal jugular vein bypass using a 6-mm polytetrafluoroethylene (PTFE) graft and banding flow reduction were performed. Postoperatively, venous hypertension resolved, DBI improved to 0.78, and steal symptoms remitted. The fistula remained patent at 6-month follow-up, demonstrating combined bypass and flow reduction as an effective salvage strategy for concurrent CTO and steal syndrome.

## Abbreviations

AVF
: arteriovenous fistula
CTO
: chronic total occlusion
DBI
: digital-brachial index
ESRD
: end-stage renal disease
PTFE
: polytetrafluoroethylene
Qa
: access flow

## Introduction

Central venous stenosis and occlusion remain significant complications in long-term hemodialysis patients, particularly in those with upper extremity arteriovenous fistulas (AVFs).^1,2)^ Repeated central venous catheterization, high-flow access, and prolonged dialysis duration contribute to progressive venous outflow obstruction, which may ultimately result in venous hypertension, access dysfunction, and steal syndrome.^1,3)^

Endovascular intervention is currently recommended as the first-line treatment for central venous lesions.^1–8)^ However, chronic total occlusion (CTO) of central veins is frequently resistant to endovascular recanalization, characterized by intimal hyperplasia, dense fibrosis, and long-segment obstruction, with low technical success and high recurrence rates.^3,6)^ When endovascular options fail, surgical alternatives become limited, especially in patients with a single remaining functional access.^7)^

In this report, we describe a case of CTO of the right brachiocephalic vein in a long-term hemodialysis patient with a high-flow right brachiocephalic AVF, complicated by simultaneous venous hypertension and steal syndrome. We present a combined surgical approach—extra-anatomic bypass with concomitant flow modulation—as a definitive salvage strategy for this hemodynamically complex scenario.

## Case Report

A 65-year-old patient with end-stage renal disease (ESRD) had been on maintenance hemodialysis for 5 years. The patient had a right brachiocephalic AVF that had been functional for 3 years and served as the sole remaining vascular access, having exhausted all other access sites. Dialysis adequacy was preserved with a measured access flow (Qa) of approximately 2500 mL/min as assessed by duplex ultrasonography.

**Fig. 1:**
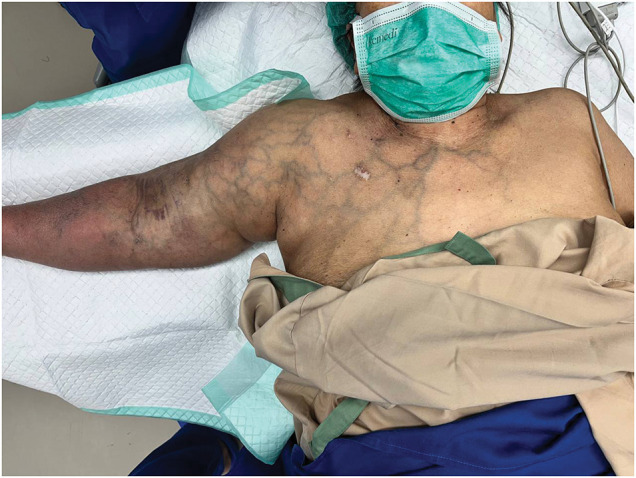
Preoperative clinical photograph demonstrating severe venous hypertension secondary to CTO of the right brachiocephalic vein. Prominent, tortuous, dilated superficial collateral veins are visible extending from the right upper limb across the entire anterior chest wall, a hallmark presentation of central venous outflow obstruction. CTO: chronic total occlusion

The patient presented with a 3-month history of progressive right upper limb edema, prominent superficial chest wall veins, and symptoms consistent with venous hypertension—a recognized manifestation of central venous outflow obstruction^1,2)^ (**Fig. 1**). Additionally, the patient developed dialysis access–induced steal syndrome grade 2 according to the Tordoir classification, characterized by hand coolness, paresthesia, and pain during dialysis sessions, but without rest pain or tissue loss.^1,8)^ The diagnosis of steal syndrome was established by clinical assessment and confirmed by digital-brachial index (DBI) measurement, which yielded a value of 0.42 (normal ≥0.6). On physical examination, prominent venous dilatation was observed over the right anterior chest wall, consistent with extensive collateral venous formation.

**Fig. 2:**
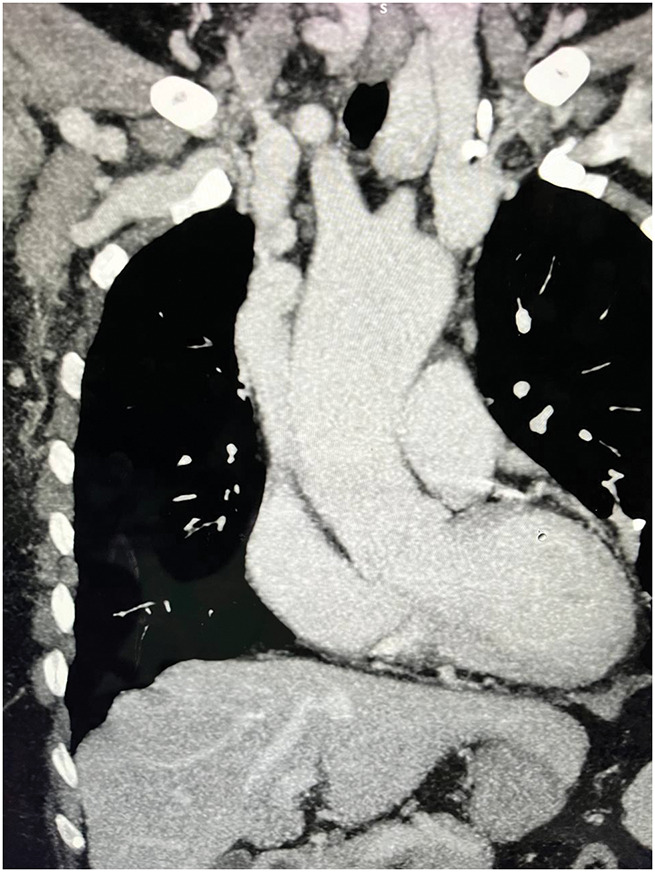
Venous-phase contrast-enhanced CTA demonstrating CTO of the right brachiocephalic vein. The right brachiocephalic vein is not opacified on venous-phase imaging, consistent with complete fibrotic occlusion. The superior vena cava and left brachiocephalic vein demonstrate normal contrast enhancement. Extensive mediastinal collateral formation is present, indicative of long-standing central venous occlusion. CTO: chronic total occlusion; CTA: computed tomography angiography

Duplex ultrasonography demonstrated absent flow in the right brachiocephalic vein, with high-resistance waveforms and elevated venous pressures in the ipsilateral upper extremity. Contrast-enhanced computed tomography angiography (CTA) confirmed CTO of the right brachiocephalic vein, approximately 4 cm in length, with non-opacification of the right brachiocephalic vein on venous-phase imaging and extensive collateral formation, findings characteristic of long-standing central venous occlusion^3,6)^ (**Fig. 2**). Multiple endovascular attempts to traverse the occlusion with hydrophilic guidewires and support catheters were unsuccessful due to dense fibrotic obstruction.^6)^ Alternative access creation was not feasible, given exhausted bilateral upper extremity and central venous options.

**Fig. 3:**
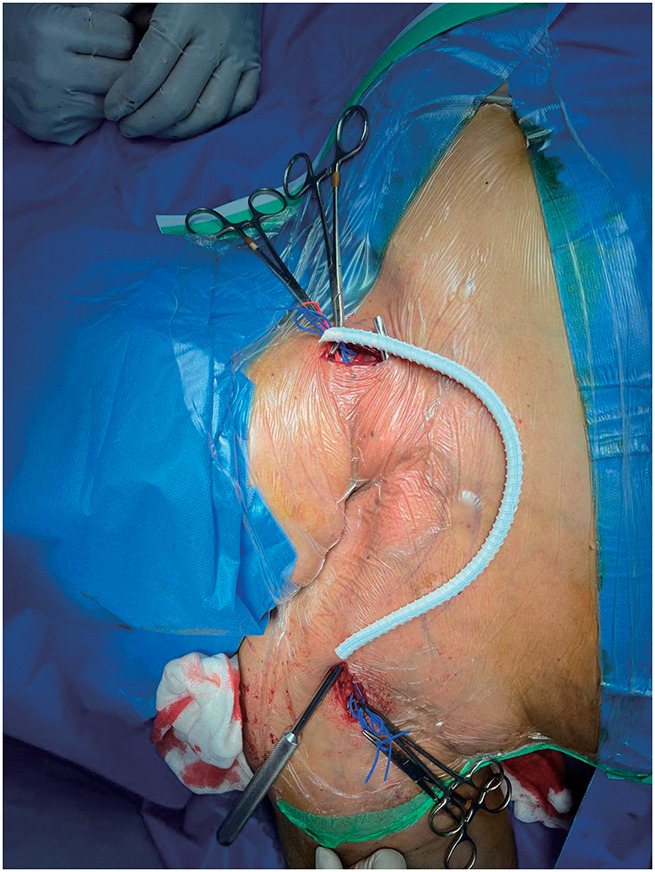
Intraoperative photograph demonstrating the completed extra-anatomic cross-neck bypass. The 6-mm ringed PTFE graft is tunneled subcutaneously across the anterior neck. The proximal anastomosis is placed end-to-side at the right cephalic arch at the deltopectoral groove (upper anastomotic site), confirming the cephalic arch—the terminal curved segment of the cephalic vein at its junction with the axillary vein—as the inflow site, distinct from the axillary vein itself. The distal anastomosis is directed toward the left IJV (lower anastomotic site). Blue sutures mark both anastomotic sites. PTFE: polytetrafluoroethylene; IJV: internal jugular vein

**Fig. 4:**
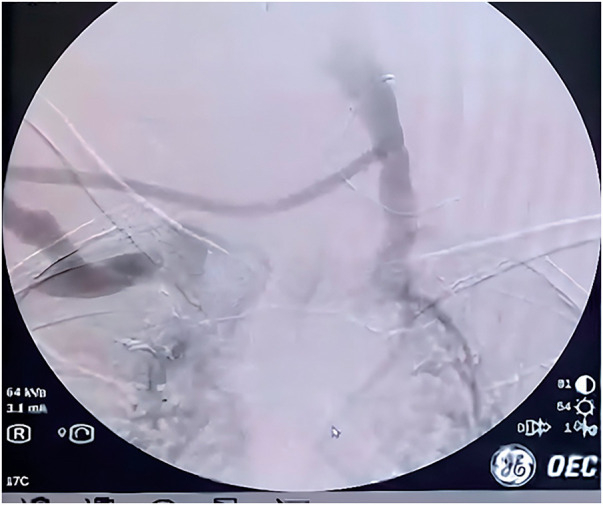
Intraoperative venography confirming patency of the cross-neck venous bypass graft. Contrast is seen flowing through the 6-mm PTFE graft from the right cephalic arch inflow, traversing the subcutaneous anterior neck tunnel, with prompt drainage toward the left IJV, confirming technical success of the bypass procedure. PTFE: polytetrafluoroethylene; IJV: internal jugular vein

Given the absence of endovascular solutions and the necessity to preserve the last functioning access, an extra-anatomic cross-neck venous bypass was planned. Under general anesthesia, with the patient in a supine position and the neck extended, the right cephalic arch was exposed through a deltopectoral incision and the left internal jugular vein (IJV) was exposed through a left cervical incision. A subcutaneous tunnel was created across the anterior neck. A 6-mm ringed polytetrafluoroethylene (PTFE) graft was anastomosed end-to-side to the right cephalic arch and end-to-side to the left IJV, with a tunnel length of approximately 18 cm (**Fig. 3**). Intraoperative venography confirmed contrast flow through the bypass graft with prompt drainage toward the left IJV, confirming technical success (**Fig. 4**). Intraoperative completion duplex confirmed graft patency with a peak systolic velocity of 180 cm/s.

Concomitant flow reduction was performed using the banding technique. A PTFE patch was used to create a controlled stenosis at the juxta-anastomotic segment of the brachial artery inflow, reducing the fistula diameter under intraoperative duplex guidance. The banding was calibrated to achieve a target Qa of 1000–1400 mL/min. Final intraoperative Qa was measured at approximately 1200 mL/min.

Postoperatively, the patient experienced marked improvement in venous hypertension, with complete resolution of arm edema within 2 weeks. Distal hand perfusion improved significantly, with DBI increasing from 0.42 preoperatively to 0.78 at 4 weeks postoperatively. Steal symptoms resolved completely. The AVF remained patent and functional for hemodialysis throughout 6 months of follow-up, with no episodes of graft thrombosis, infection, or access-related complications.

## Discussion

We acknowledge that extra-anatomic venous bypass for central venous obstruction is an established technique.^7)^ However, the scientific significance of this case lies not in the bypass procedure itself, but in the simultaneous management of 2 hemodynamically opposing pathologies—CTO causing venous hypertension, and high-flow dialysis access–induced steal syndrome—in a patient with no remaining access alternatives. To our knowledge, this specific combination of outflow reconstruction and inflow modulation performed concurrently in the setting of CTO with steal syndrome and a single remaining access has not been previously described as a defined management strategy. This case therefore provides practical surgical guidance for a rare but clinically critical scenario.

The coexistence of CTO and steal syndrome in the same access presents a unique surgical dilemma. Relieving outflow obstruction alone, without addressing the high inflow state, risks perpetuating or worsening steal symptoms: decompression of the venous outflow may paradoxically increase net arterial inflow demand, exacerbating distal ischemia. Conversely, flow reduction alone would not address venous hypertension or protect the access from occlusion-related dysfunction. The decision to combine both interventions in a single operative session required careful preoperative hemodynamic assessment and intraoperative duplex-guided calibration, and resulted in objective normalization of both venous outflow and distal perfusion.

Endovascular intervention, including percutaneous transluminal angioplasty with or without stenting, remains the first-line approach for central venous obstruction.^1,3)^ However, CTO is associated with substantially lower technical success rates compared to stenotic lesions, owing to the dense fibrotic core that resists wire crossing.^3,6)^ In our patient, multiple attempts failed, confirming the appropriateness of surgical escalation. In a patient with a single remaining access, the threshold for surgical intervention must account for the catastrophic consequence of access loss—namely, the absence of any further dialysis option.

The cephalic arch was selected as the bypass inflow site because it provides direct continuity with the AVF outflow tract, minimizing anastomotic complexity and preserving the native fistula configuration.^7)^ The cephalic arch refers to the terminal curved segment of the cephalic vein as it turns medially to drain into the axillary vein at the deltopectoral groove—distinct from and medial to the axillary vein itself. As demonstrated in the intraoperative photograph (**Fig. 3**), the PTFE graft emerges from the deltopectoral groove region, confirming the cephalic arch as the inflow site. Routing the bypass to the contralateral IJV leverages the patent contralateral central venous system while circumventing the occluded right brachiocephalic vein, as previously described by Illig et al.^7)^

The banding technique for flow reduction was chosen for its established safety profile, ease of intraoperative titration under duplex guidance, and reversibility if over-reduction occurs.^1,8)^ The final Qa of 1200 mL/min achieved adequate dialysis adequacy while resolving steal, as confirmed by normalization of the DBI from 0.42 to 0.78.

This case contributes to the evidence that combined outflow reconstruction and inflow modulation can successfully salvage complex hemodialysis access scenarios. Surgeons managing patients with concurrent CTO and steal syndrome should perform comprehensive preoperative hemodynamic evaluation, plan for both pathologies simultaneously, and use intraoperative flow monitoring to optimize outcomes.

## Conclusion

In this patient with CTO of the right brachiocephalic vein and no viable endovascular or access alternatives, simultaneous extra-anatomic cross-neck cephalic arch-to-contralateral IJV bypass using a 6-mm PTFE graft and concomitant banding flow reduction successfully preserved the last functional hemodialysis access. Venous hypertension resolved, steal symptoms remitted, and the fistula remained patent at 6-month follow-up. The novelty of this case lies in the combined surgical management of 2 hemodynamically opposing pathologies in a single session—a strategy that should be considered in carefully selected patients when endovascular options have been exhausted and access loss is not an acceptable outcome.
